# Impact of deprescribing intervention on potentially inappropriate medications and clinical outcomes among hospitalized older adults in Malaysia: a randomized controlled trial (REVMED RCT) protocol

**DOI:** 10.1186/s40545-023-00621-5

**Published:** 2023-10-03

**Authors:** Chee Tao Chang, Siew Li Teoh, Wee Kooi Cheah, Pei Jia Lee, Muhammad Azuan Azman, Shiau Hui Ling, Angie Su Ching Chuah, Noor Hamizah Sabki, Doris George, Hoey Lin Oh, Jing Yi Goh, Siew Huang Lee, Wai Keng Foong, Jason Choong Yin Lee, Huan Keat Chan, Lee Rhui Teoh, Xin Jie Lim, Philip Rajan, Shaun Wen Huey Lee

**Affiliations:** 1 Clinical Research Centre, Hospital Raja Permaisuri Bainun, Ministry of Health Malaysia, Ipoh, Perak Malaysia; 2 School of Pharmacy, Monash University Malaysia, Subang Jaya, Malaysia; 3 Clinical Research Centre, Hospital Taiping, Ministry of Health Malaysia, Taiping, Malaysia; 4 Pharmacy Department, Hospital Raja Permaisuri Bainun, Ministry of Health Malaysia, Ipoh, Malaysia; 5 Pharmacy Department, Hospital Seri Manjung, Ministry of Health Malaysia, Seri Manjung, Malaysia; 6 Pharmacy Department, Hospital Taiping, Ministry of Health Malaysia, Taiping, Malaysia; 7 Pharmacy Department, Hospital Slim River, Ministry of Health Malaysia, Slim River, Malaysia; 8 Pharmacy Department, Hospital Kuala Kangsar, Ministry of Health Malaysia, Kuala Kangsar, Malaysia; 9 Pharmacy Department, Hospital Batu Gajah, Ministry of Health Malaysia, Batu Gajah, Malaysia; 10 Perak Pharmaceutical Services Division, Ministry of Health Malaysia, Tanjung Rambutan, Malaysia; 11 Clinical Research Centre, Hospital Sultanah Bahiyah, Ministry of Health Malaysia, Alor Setar, Malaysia; 12 Pharmacy Department, Hospital Sungai Siput, Ministry of Health Malaysia, Sungai Siput, Malaysia; 13 Department of Medicine, Hospital Taiping, Ministry of Health Malaysia, Taiping, Malaysia

**Keywords:** Polypharmacy, Deprescribing, Potentially inappropriate medications, Geriatrics, Fall, Readmission, Quality of life

## Abstract

**Background:**

Polypharmacy and the use of potentially inappropriate medications (PIMs) are prevalent among older patients admitted to hospitals, posing a heightened risk of adverse drug events. This trial aims to evaluate the effectiveness of a pharmacist-led deprescribing intervention in reducing medications, PIM and improving clinical outcomes, using the locally developed Malaysian Potentially Inappropriate Prescribing Screening tool in Older Adults (MALPIP).

**Methods:**

This is an 18-month cluster-randomized, open-label, parallel-arm controlled trial conducted at 14 public hospitals in the Perak state of Malaysia. Patients aged 60 and above, who have at least one medication and one comorbidity are eligible. A stratified-cluster randomization design is employed, with 7 hospitals assigned to the control arm and 7 hospitals assigned to the intervention arm. The MALPIP screening tool will be used in the intervention group to review the medications. If PIM is detected, the pharmacists will discuss with doctors and decide whether to stop or reduce the dose. The primary outcomes of this trial are the total number of medications and number of PIM. The secondary outcomes include fall, emergency department visits, readmissions, quality of life and mortality. Outcomes will be measured during enrolment, discharge, 6, 12, and 18 months.

**Discussion:**

This REVMED trial aims to test the hypothesis that a pharmacist-led deprescribing intervention initiated in the hospital will reduce the total number of medications and PIM 18 months after hospital discharge, reducing fall, emergency department visits, readmissions, mortality and lead to improvement in quality of life. Trial findings will quantify the clinical outcomes associated with reducing medications and PIM for hospitalized older adults with polypharmacy.

*Trial registration number:* This trial was prospectively registered at clinicaltrials.gov (NCT05875623) on the 25th of May 2023. NCT05875623 Clinicaltrials.gov URL: NCT05875623 registered on 25th July 2023.

## Introduction

Globally, the world is ageing. The United Nations estimates that by 2050, one in six people in the world will be over age 65 (16%), up from one in 11 in 2019 (9%) [1]. While this can be considered one of the successes of the twenty-first century, older individuals are more likely to experience multiple health issues, thus requiring more medication than their younger counterparts. This poses another problem for many health systems, as the prescription of various medications can lead to drug-related problems, including polypharmacy, the use of potentially inappropriate medications, and challenges with medication adherence [2, 3]

Polypharmacy, which is commonly defined as regular use of five or more prescription drugs [4] increases the likelihood of an adverse drug event (ADE) and hospitalization [5, 6]. In addition, polypharmacy is also associated with an increased risk of multiple falls [7, 8] and the use of potentially inappropriate medications (PIM) among older adults [9].

Deprescribing has been suggested to reduce the number of medications and mitigate the potential for adverse drug events resulting from inappropriate medication use in older adults [10]. To guide deprescribing, several explicit tools have been developed to identify potentially inappropriate prescribing in older adults. The most widely used explicit PIM tool is the American Geriatrics Society Beers' criteria for Potentially Inappropriate Medication Use in Older Adults. Another commonly used tool is the Screening Tool of Older Person's Prescriptions (STOPP) and the Screening Tool to Alert doctors to Right Treatment (START) to facilitate the detection of potential omission and commission errors in prescribing [11, 12].

Evidence from randomized controlled trials has demonstrated the effectiveness of using explicit PIM criteria in reducing PIM. Systematic review of randomized controlled trials demonstrated that deprescribing reduced medication burden and positively impacts health-related quality of life, costs, and hospitalization [13]. Deprescribing interventions also reduced medication errors and potential drug–drug interactions [14–16]. While studies have demonstrated the effectiveness of explicit PIM criteria in improving older patients’ outcomes, most relied on the Western PIM criteria, such as START-STOPP and Beers criteria [17–19].

In the context of deprescribing interventions, it is crucial to consider the diverse population demographic, pharmacogenomics, prescribing practices, and cultural factors that exist within different regions [20–22]. By taking these factors into consideration, the intervention is better aligned with the specific needs and characteristics of the target population. The adoption of a tailored approach in this trial acknowledges the distinct differences that may arise within the Asian population, particularly in a country like Malaysia.

## Methods

### Aim of the trial

This trial aims to assess the effectiveness of a pharmacist-led deprescribing intervention using the locally developed Malaysian Potentially Inappropriate Prescribing Screening tool in Older Adults (MALPIP) in reducing chronic medications, PIMs and improving clinical outcomes among older adults who have recently been hospitalized.

### Outcome measures

The primary outcome of the trial is the total number of chronic medications and PIM taken by older adults at five time-points (admission, discharge, 6 months, 12 months and 18 months post-randomization).

The secondary outcomes include fall events, number of emergency department visits, number of ADE-related and all-cause readmissions to the hospital, and mortality at 6 months, 12 months, and 18 months post-randomization. The total number of pharmacists' interventions and acceptance by doctors will be measured at discharge. The quality of life will be measured at admission, discharge, 6 months, 12 months, and 18 months post-randomization (Table 1).

**Table 1:** Primary and secondary outcomes, instruments, and time-points

	Instruments	Time-points
Primary outcome		
1. PIM	MALPIP	Admission, discharge, 6 months, 12 months and 18 months
2. Chronic medications	Medication prescribed continuously for more than 28 days prior to the assessment day	Admission, discharge, 6 months, 12 months and 18 months
Secondary outcome		
1. Recommendations made by pharmacists and accepted by physicians	Pharmacists’ pharmacotherapy review form and patients’ case note	Discharge
2. Fall event	Medical records and self-reported using ICD-10 codes	discharge, 6 months, 12 months and 18 months
3. Emergency Department (ED) visit	Medical records and self-reported	6 months, 12 months and 18 months
4. ADE-related and all-cause readmissions	Medical records and self-reported	6 months, 12 months and 18 months
5. Quality of life	EQ-5D-5L	Admission, discharge, 6 months, 12 months and 18 months
6. Survival status	National Registration Department database	discharge, 6 months, 12 months and 18 months

### Overview of trial design

This is an 18-month cluster-randomized, open-label, multicenter, parallel-arm controlled trial at 14 public hospitals in the Perak state of Malaysia among older patients (≥ 60 years old). The deprescribing interventions will be led by ward pharmacists who had at least 5 years of clinical pharmacy experience.

The ward pharmacists undertake a comprehensive review of the patient's medication before performing the deprescribing interventions.

### Trial setting

**Fig. 1:**
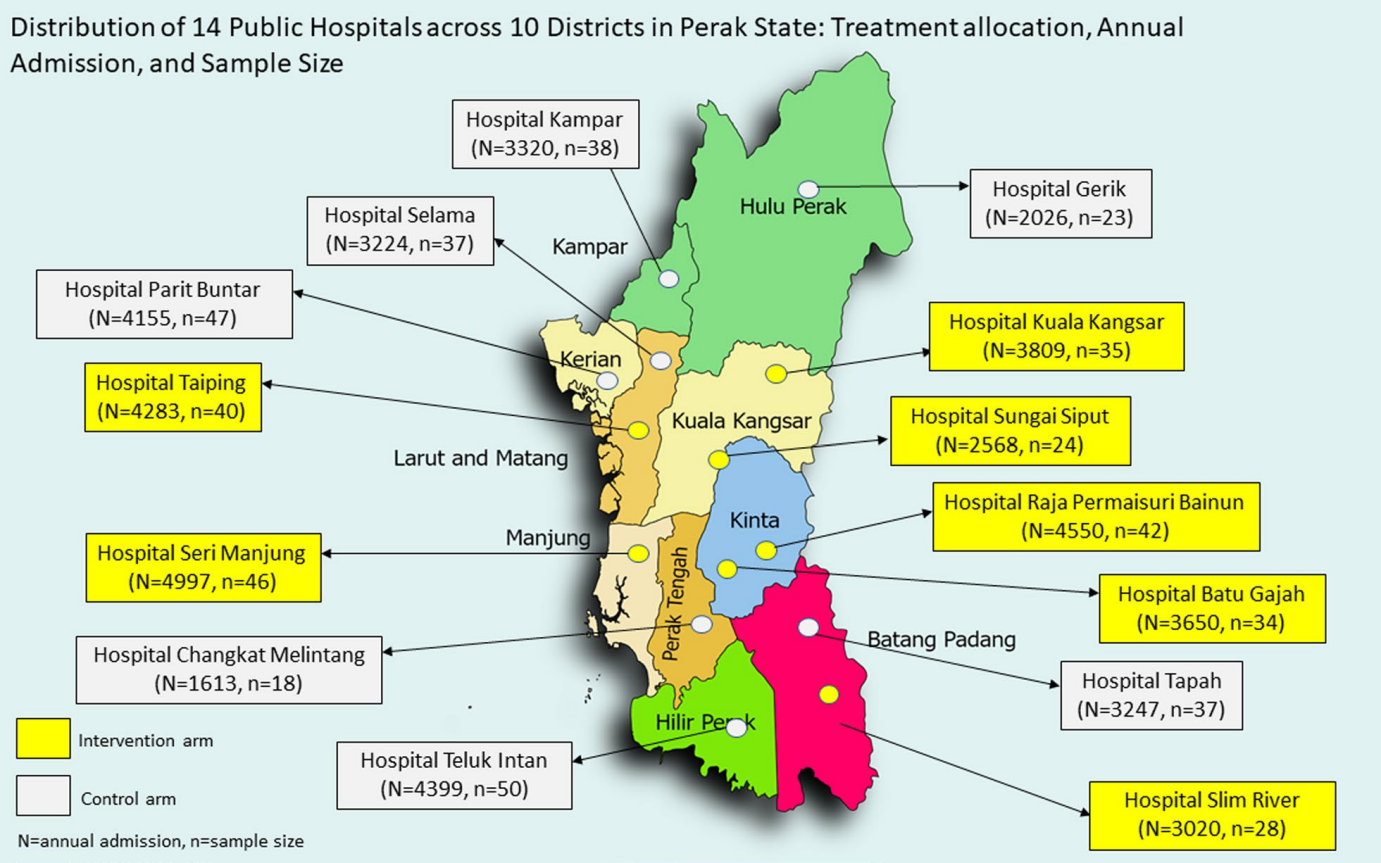
Distribution of 14 public hospitals across 10 districts in Perak State: treatment allocation, annual admission, and sample size

The trial will be conducted in the state of Perak, which has one of the highest proportion (8.9%) of older adults aged 65 years and above in Malaysia [23]. The state is served by 14 public hospitals spread across the 10 administrative districts. In this study, seven hospitals will be randomized to intervention with the remaining seven hospitals serving as the control group (Fig. 1). In order to ensure a representative population is recruited, both general medicine wards or geriatric wards will be selected from each hospital, from both female and male wards.

### Randomization

A stratified-cluster randomization design will be adopted for this trial, with the 14 hospitals serving as the unit of randomization. This design was chosen specifically to address the potential "contamination" effect that may arise among individuals within the same ward. The cluster design effectively controls for the potential "contamination" effect that may arise among individuals (pharmacists, doctors, patients) within the same ward, where changes in one individual's behaviour may have the potential to influence others. Furthermore, this approach offers the advantage of significant time savings compared to individual randomization. The principal investigator performed the randomization.

Given that larger and smaller hospitals may have different administrative capacities, it was crucial to ensure a balanced distribution of patients across both intervention and control arms. To achieve this, trial sites were stratified and clustered based on the number of yearly admissions in each hospital as follows:

i. Hospitals with 4001–5000 yearly admissions (total of 5 hospitals; intervention: 3; control: 2)
ii. Hospitals with 3001–4000 yearly admissions (total of 6 hospitals; intervention: 3; control: 3)
iii. Hospitals with 1500–3000 yearly admissions (total of 3 hospitals; intervention: 1; control: 2).

This approach ensures a balanced and comparable distribution of patients in both the intervention and control groups.

### Sample size calculation

Sample size was calculated based on the primary outcome: the reduction in the mean number of PIM in discharge. According to Wehling et al. [24], the mean number of PIMs at discharge for the intervention group was 0.15, while the mean number of PIMs at discharge for the control group was 0.28 [24]. For an alpha risk of 0.05 and a 90% power, standard deviation 0.39, it was estimated that 189 patients would be needed per arm (378 patients in total) to detect a 0.13 reduction in the mean number of PIM [24]. Projecting a subject dropout rate of 25%, the total number of subjects required will be 500, or 250 per arm. The number of samples per trial site will be stratified based on yearly admissions. Sample size was calculated using Power and Sample size online calculator, compare two proportions, two-sample, two-sided equality test [25].

### Flow of study subjects

**Fig. 2:**
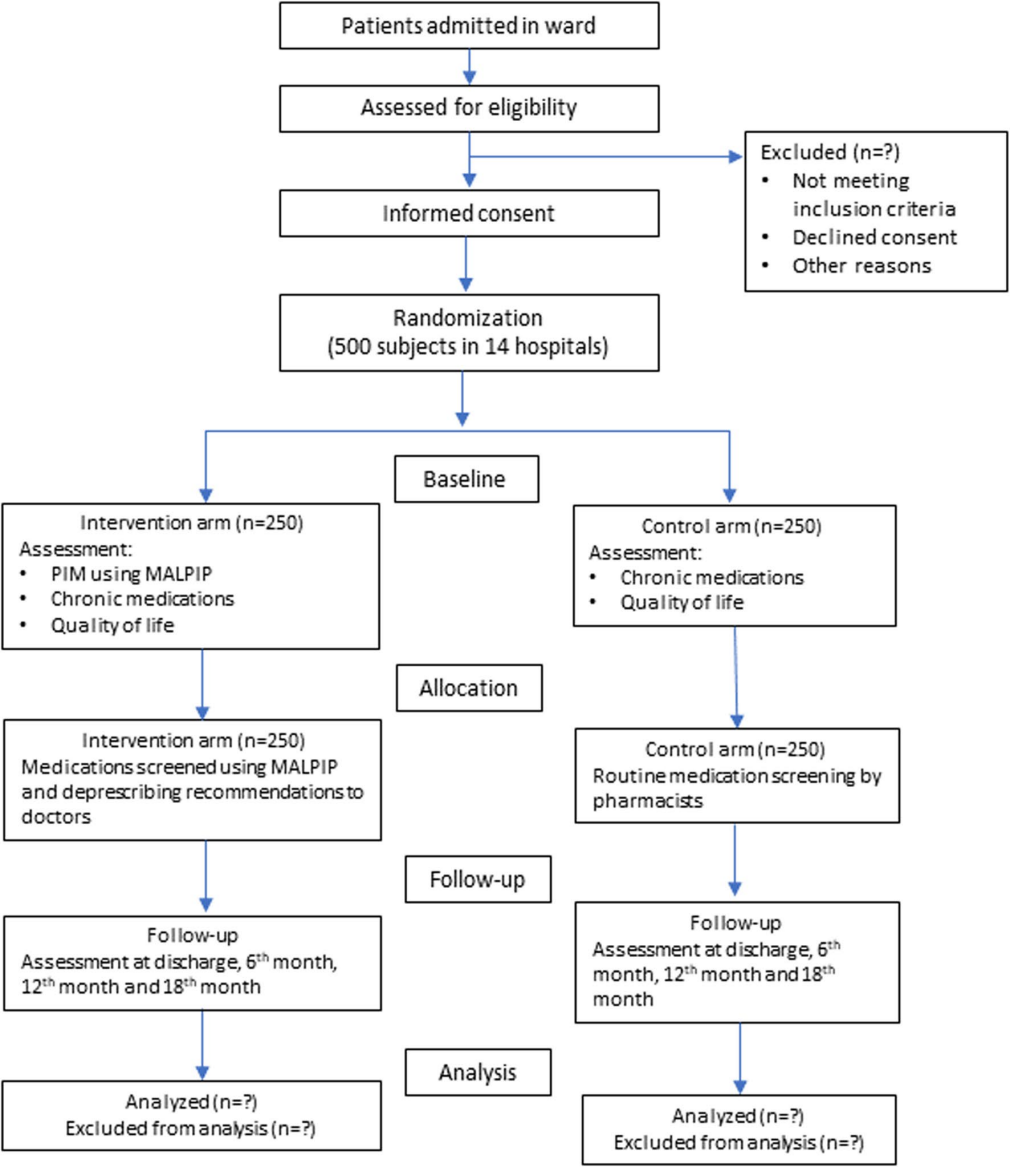
Flow of study subjects

Figure 2 describes the study subject’s flow, and the subsequent parts of this protocol outline each step in detail.

### Recruitment

At each study site, pharmacist will actively identify patients who meet the eligibility criteria. Participants will be provided a detailed explanation regarding the purpose and procedures of the trial. Patients who express their agreement to participate will be required to provide a written informed consent before enrolment. For patients with neurological or psychiatric illnesses, the consent and signature of their caregivers or legal representatives will also be obtained.

Participants will be recruited if they meet the following inclusion criteria: (1) patients aged ≥ 60 years old; (2) taking more than five regular drugs at the time of admission; (3) taking at least one chronic medical condition using International Classification of Diseases, Tenth Revision, Clinical Modification (ICD-10-CM); (4) can understand and speak Malay, English, or Mandarin language.

Patients will be excluded if they: (1) are admitted for end-of-life care; (2) diagnosed with terminal illness; (3) diagnosed with active cancer; (4) participated in another drug trial; (5) refused or unable to give consent; (5) visited the Emergency Department without admission to ward; (6) readmitted and have been previously enrolled in the trial.

### Control arm

All participating patients will receive the standard routine care typically provided at each respective site. This includes preadmission medication reconciliation and medication review routinely conducted by the pharmacists. Adherence to routine practices in the control arm is integral to ensuring comprehensive patient care and align with the standard procedures implemented within the healthcare setting.

### Intervention arm

**Fig. 3:**
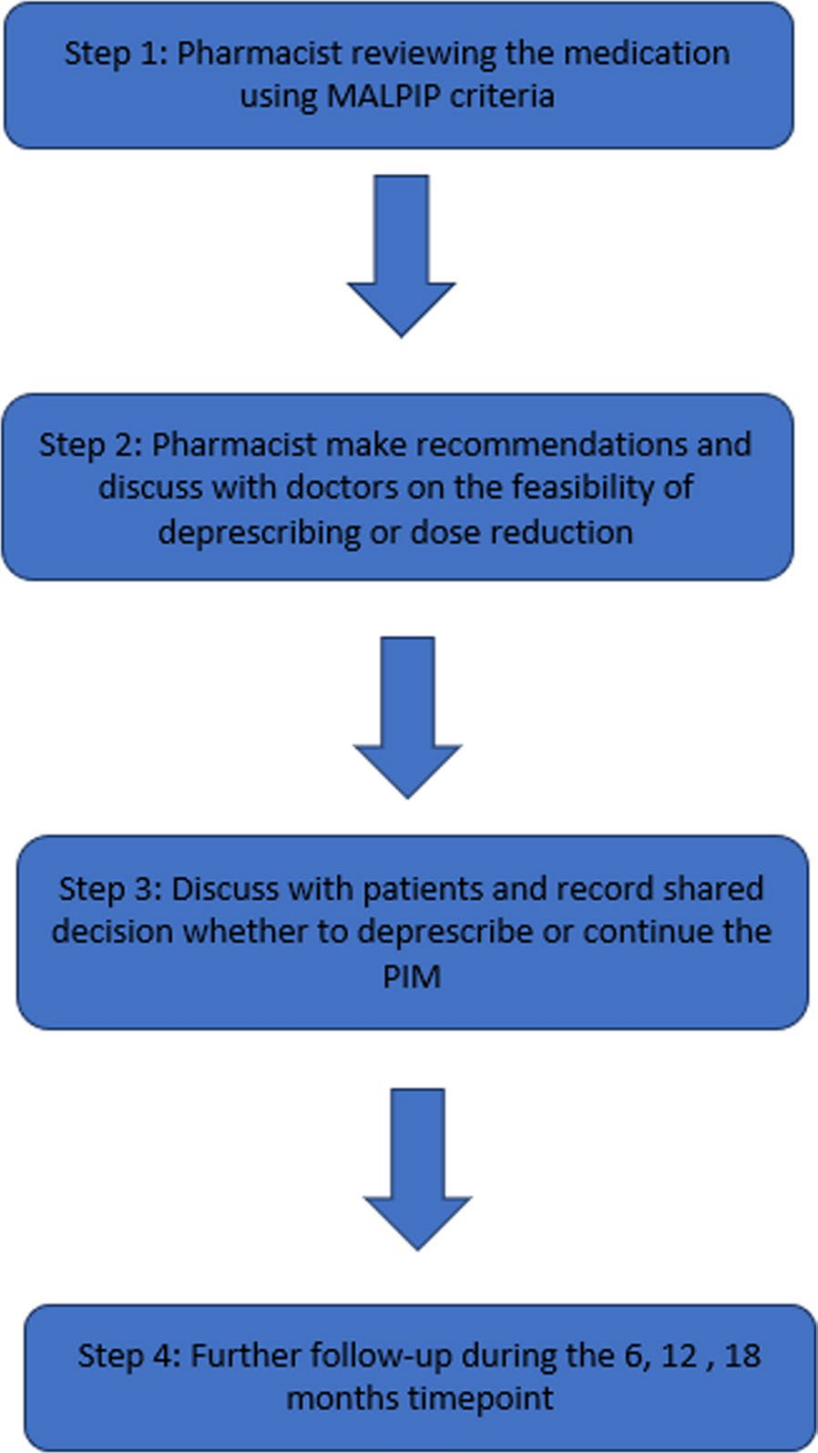
Four-step pharmacist-led deprescribing framework

In the intervention group, a four-step deprescribing process will be employed, whereby: i) pharmacist review using MALPIP criteria to detect PIM on all the admission and discharge medications; ii) discussion with doctors for deprescribing decisions; iii) discussion with patients and documentation of shared decisions; iv) follow-up patients (Fig. 3).

Pharmacists will conduct deprescribing interventions and engage in discussions with doctors during daily medical ward rounds, where deprescribing decisions will be made based on the pharmacists' suggestions and the attending doctors' judgment. Following this, the pharmacists will communicate the deprescribing proposals to patients, and document the decision whether it was approved or rejected by the physicians. Patient outcomes, including medication usage, quality of life, falls, and readmission rates, will be assessed throughout the study. The complete MALPIP list can be found at https://sites.google.com/moh.gov.my/malpip/home and the mobile application can be downloaded from Google Play Store: https://play.google.com/store/apps/details?id=pack.geridea.

To ensure standardization, pharmacist serving each of the medical ward will receive a half-day face-to-face training session on the intervention. To ensure data integrity, the principal investigator will perform data validation by reviewing a randomly selected 20% of the study data, focusing on the accuracy of PIM coding.

### Blinding

Blinding is not possible due to the nature of the intervention. As such, participants assigned to the intervention group will be informed regarding the intervention. In the control group, all participants, physicians, and ward pharmacists will continue to receive and provide their usual care without any knowledge of the intervention. Routine pharmacist medication review services will address significant drug errors identified, which will be communicated to the physicians.

### Measurements

Table 1 describes the outcomes that will be measured in this trial. Falls will be classified using ICD-10 codes based on patient-reported data. The number of emergency department visits and readmissions to hospitals will be based on hospital medical records and patients' reported data. Survival status at 6, 12 and 18 months will be retrieved from the National Registration Department database at the end of the trial.

Chronic medications will include drugs prescribed consistently for more than 28 days prior to the assessment day (Bayliss et al. 2020), excluding certain medication categories such as toxoids, vaccines, local anaesthetics-parenteral, antiseptics and disinfectants, and antidotes, as per the 2-digit GPI codes classification.

To assess the subject's quality of life, the Malaysian-validated version of EQ-5D-5L will be employed [26], which offers greater robustness compared to EQ-5D-3L [27, 28]. The face-to-face and telephone interview versions of the questionnaire are obtained from the EuroQoL office, and they are available in Malay, Chinese, and English languages. The choice of administration will be based on the subject's preference.

### Data analysis

All analyses will adhere to the intention-to-treat principle, complemented by per-protocol analysis as sensitivity analyses. Descriptive statistics will be employed to analyse the baseline characteristics of the patients. The change in the mean number of chronic medications and potentially inappropriate medications (PIM) across four time-points will be assessed using repeated measures ANOVA. Independent t-tests will be utilized to compare the number of emergency department (ED) visits and hospitalizations between the control and intervention groups. A p-value of less than 0.05 will be deemed statistically significant. IBM SPSS Statistics for Windows version 24 (IBM Corporation, NY, USA) will be employed for statistical analyses.

### Ethics approval

Ethics approval was obtained from the Medical Research Ethical Committee (MREC): 22-02454-CQA. Study protocol was registered in Monash University Human Research Ethics Committee (Project number: 39249).

## Discussion

This trial aims to assess the feasibility, safety, and effectiveness of a locally developed deprescribing intervention utilizing explicit criteria (MALPIP) for older adults during hospital admission and discharge. Previous studies have demonstrated the effectiveness of deprescribing interventions using explicit criteria in reducing potentially inappropriate medications (PIM), chronic medications, adverse drug reactions, and drug interactions [14–16].

However, the existing evidence is limited regarding clinically meaningful outcomes such as falls, readmissions, and mortality. A recent systematic review found inconclusive evidence regarding the impact of deprescribing fall-risk-increasing drugs on reducing falls [17]. Similarly, there is insufficient definitive evidence on the effect of deprescribing interventions on improving quality of life, reducing hospital admissions, or mortality rates [19, 29–31].

This trial aims to fill the existing knowledge gap by investigating the impact of deprescribing interventions on falls, quality of life, readmission, and mortality, which were previously not conclusively established. Firstly, the effectiveness of deprescribing on long-term clinical outcomes will be assessed over an 18-month period, yielding valuable data on the sustainability of the intervention's impact. Secondly, the impact of deprescribing on patients' quality of life will be addressed, aligning with patient-centred care and holistic healthcare. Lastly, the utilization of locally developed deprescribing criteria (MALPIP) will enhance the relevance, acceptance, and simplicity of the deprescribing interventions within the local healthcare setting.

### Implications

The outcomes of this trial are highly relevant for policymakers as they will shed light on the feasibility of deprescribing interventions in older adults. Furthermore, the findings will contribute to the development of national deprescribing guidelines in Malaysia and increase awareness among healthcare professionals regarding appropriate medication prescribing, management of polypharmacy, and identification of potential drug-related issues in the older population.

Moreover, this trial serves as a valuable resource for policymakers, offering insights into the factors that contributed to the success of the deprescribing intervention as well as the limitations encountered during implementation. Such information is crucial for optimizing resources and tailoring policies, facilitating evidence-based decision-making when implementing deprescribing interventions in an Asian context like Malaysia.

## Data Availability

All data to reproduce the tables and figures in the manuscript and Supplementary Information can be obtained with reasonable request from the corresponding author.
